# Role of Structure-Based Changes due to Somatic Mutation in Highly Homologous DNA-Binding and DNA-Hydrolyzing Autoantibodies Exemplified by A23P Substitution in the VH Domain

**DOI:** 10.1155/2012/683829

**Published:** 2012-11-11

**Authors:** A. V. Kozyr, A. V. Kolesnikov, A. E. Khlyntseva, A. G. Bogun, G. A. Savchenko, I. G. Shemyakin, A. G. Gabibov

**Affiliations:** ^1^State Research Center for Applied Microbiology and Biotechnology, Serpuhov District Obolensk 142279, Russia; ^2^Institute of Immunological Engineering, Lyubuchany 142380, Russia; ^3^Shemyakin & Ovchinnikov Institute of Bioorganic Chemistry, Russian Academy of Sciences, Miklukho-Maklaya Street 16/10, Moscow 117997, Russia

## Abstract

Anti-DNA autoantibodies are responsible for tissue injury in lupus. A subset of DNA-specific antibodies capable of DNA cleavage can be even more harmful after entering the living cells by destroying nuclear DNA. Origins of anti-DNA autoantibodies are not fully understood, and the mechanism of induction of DNA-cleaving activity remains speculative. The autoantibody BV04-01 derived from lupus-prone mouse is the only DNA-hydrolyzing immunoglobulin with known 3D structure. Identification and analysis of antibodies homologous to BV04-01 may help to understand molecular bases and origins of DNA-cleaving activity of autoantibodies. BLAST search identified murine anti-DNA autoantibody MRL-4 with sequences of variable region genes highly homologous to those of autoantibody BV04-01. Despite significant homology to BV04-01, not only MRL-4 had no DNA-cleaving activity, but also reversion of its unusual P23 mutation to the germline alanine resulted in a dramatic loss of affinity to DNA. Contrary to this effect, transfer of the P23 mutation to the BV04-01 has resulted in a significant drop in DNA binding and almost complete loss of catalytic activity. In the present paper we analyzed the properties of two homologous autoantibodies and mutants thereof and discussed the implications of unusual somatic mutations for the development of autoantibodies with DNA-binding and DNA-hydrolyzing activity.

## 1. Introduction

Anti-DNA autoantibodies are known as important factors of tissue injury in autoimmune diseases, such as SLE [1]. Immune complexes containing anti-DNA antibodies form tissue deposits primarily in kidney that cause apoptotic cell death and severe tissue injury [2, 3].

Nucleic acid cleaving antibodies represent a subset of autoantibodies, capable of single-stranded and double-stranded DNA and RNA hydrolysis [4, 5]. Anti-DNA autoantibodies penetrate cellular membrane and localize to the nuclei of living cells [6]. Furthermore, certain DNA-cleaving antibodies may also penetrate living cells causing apoptosis in caspase-dependent manner, presumably by introducing nicks in nuclear DNA [7]. Cell death due to entry of DNA-hydrolyzing antibodies can contribute to tissue injury observed in autoimmune diseases. 

Despite decades of research, complete picture of development of DNA-binding and DNA-hydrolyzing activities by autoantibodies is lacking. Acquisition of affinity and specificity of autoantibodies to DNA is believed to proceed via antigen-driven selection on the background of impaired censoring mechanisms, which are normally deleting or silencing autoreactive B-cell clones at the healthy state [1–3]. A long-standing paradigm, which holds that nucleosomes represent the primary (auto)antigen-inducing anti-DNA antibodies, is questioned by the observations that foreign DNA-protein complexes can also serve as efficient antigens for induction of anti-DNA antibodies that cause lupus-like nephritis with proteinuria and glomerular IgG deposits [8, 9]. Other mechanisms such as molecular mimicry may also participate in the induction of anti-DNA autoantibodies [10]. At present, it is not known, if impairment of immune system censorship normally deleting autoreactive clones is required for maturation of high-affinity anti-DNA antibodies. Antibodies against foreign DNA-protein complexes as well as antibodies with DNA specificity induced by other hitherto unknown mechanisms can contribute to the development of autoimmune pathologies.

While studying murine DNA-cleaving autoantibody BV04-01 [11, 12], we identified its close homolog anti-DNA antibody MRL-4 [13]. Heavy chain of MRL-4 contained an unusual somatic mutation and replacement of germline Ala-23 by a proline. In this study, we demonstrated that a single mutation not being involved in direct interaction with the antigen can cause profound effect on the binding and catalytic properties of DNA-specific autoantibody.

## 2. Materials and Methods

### 2.1. Chemicals, Materials, Bacterial Strains, and Vector DNA

Unless stated otherwise, chemicals were purchased from Sigma. Bacterial growth media and media supplements were from VWR Scientific (BD Difco). The pET-22b(+) vector and *Escherichia coli *strains *BL21 *and *BL21*(*DE3*) were obtained from Novagen. *DH12S E. coli *was from Invitrogen, as well as pBluescript SK(−) plasmid DNA. Oligonucleotides were prepared by Syntol and Evrogen. All solutions used in this study were made using 18 MΩ ultrapure water from a Millipore synthesis station and sterilized by autoclaving. 

### 2.2. Cloning, Expression, and Purification of Single-Chain Antibody Fragments

The pET22b(+) vector was modified by PCR-guided mutagenesis to replace GCG (coding for Ala-20 in the pelB signal peptide) by the synonymous GCC with simultaneous disruption of the original Nco I cite. This permits cloning of single-chain antibody constructs with 5′-appended Nco I site and 3′ terminal pelB codons so that processing of pelB by periplasmic secretion system liberates native mature terminus of the antibody light chain. In addition, sequence encoding c-myc peptide (EQKLISEEDL) flanked by 5′-Xho II and 3′-Sal I recognition sites, was inserted in-frame with the downstream His6 tag into the Xho I site. 

 Single chain antibody SCA04-01, containing variable regions of BV04-01 heavy and light chain genes, was prepared earlier [12]. It was PCR-amplified using forward VKF (5′-CATTCCATGGCCGATGTTGTGATGACCCAA) and reverse VHR (5′-TTACTCGAGTGCAGAGACAGTGACCAGAGT) primers to introduce upstream Nco I and downstream Xho I cloning sites (underlined portions of primer sequences). Resulting PCR product was digested by Nco I and Xho I, and ligated with the modified Nco I-Xho I digested pET22b(+). cDNAs encoding VH and VL of DNA-binding antibody MRL-4 were amplified from corresponding RNA, isolated from hybridoma cells (kind provision of Dr. A. Theofilopoulos) using TriZol reagent. Primers for VH amplification were MRL1F (5′-AAGAGCTCTGAGGGTAAAGGCGAGGTGCAGCTTGTTGAGACT) as forward primer and VHR as reverse primer. 

 Amplified cDNA coding for the VL was then modified by appending upstream Nco I and downstream Spe I site with part of the scFv linker sequence, using forward VKF and reverse KLR (5′-AACACTAGTACCAGATTTTATTTCCAGCTTGGTCCCCGA) primers. VH cDNA was amplified using MRLF (5′-TCAACTAGTAGCGGCTCTGGTAAGAGCTCTGAGGGTAAAGGCGAGGTGCAGCTTGTTGAGACT) forward and VHR reverse primers to append downstream Xho I site and upstream Spe I site together with the rest of the linker sequence (GSTSGSGKSSEGKG) [14]. Amplified DNAs corresponding to VH and VL chains of BV04-01 and MRL-4 were digested by corresponding restriction enzymes and cloned into the modified pET22b(+) digested by Nco I and Xho I by 3-fragment ligation using Rapid Ligation DNA kit (Roche). 

 Light chain of antibody BV04-01 was prepared by joining  V*κ*  derived from SCA04-01 by PCR with VKF and KRR (5′-ATACTCGAGTTATTCAAGCTTGGTCCCCGAATACGGAACA) primers, and C*κ*, amplified from the mouse RNA (see above) using C*κ*F (5′-TCGGGGACCAAGCTTGAAATAAAA) and C*κ*R (5′-TCTCTCGAGACACTCATTCCTGTTGAAGCT) primers. V*κ* fragment was cloned into modified pET22b(+) vector between Nco I and Xho I sites, and C*κ* was next cloned downstream to V*κ* using PCR-introduced Hind III site and Xho I site.

DH12S cells were transformed by constructs encoding single-chain antibodies using electroporation, and correct clones were identified by colony PCR and DNA sequencing. Plasmids encoding BV04-01 and MRL-4 scFvs were isolated and used to transform BL21(DE3) cells. Transformants, plated at a density sufficient to form a lawn to the 2xYT medium, contained 1% agar, 1% glucose, and 100 *μ*g/mL ampicillin, were grown overnight, washed out from Petri dishes with 2xYT medium/0.5% glucose, and used to inoculate 4 liters of 2xYT medium, containing 0.1% glucose and 100 *μ*g/mL ampicillin. Cells were grown at 37°C until OD600 reached 0.9, then chilled to 20°C on ice, and IPTG was added to the culture to reach final concentration of 0.2 mM. Protein production continued for additional 10 hours at 20°C with vigorous shaking. Isolation of periplasmic scFvs was performed following earlier described protocol [15]. After growth completion, the culture was chilled on ice for 20 min and pelleted by 15 minutes of centrifugation at 3000 ×g at 4°C. The pellet was washed with the buffer containing 200 mM Tris-HCl, 20% sorbitol, and 1 mM EDTA, pH 8.0, resuspended in 50 mL of the same buffer and incubated on ice for 1 hour with occasional stirring. Cell debris was removed by centrifugation at 30000 ×g for 40 min. Supernatant was extensively dialyzed against the chelating buffer (100 mM NaCl, 50 mM Tris–HCl, and pH 8.0). Protein and DNA purification was conducted using FPLC AKTA system (GE Healthcare) with corresponding software and accessories. Clarified periplasmic fraction was loaded on the column, which has been prepacked with 10 mL of Talon chelating resin (Clontech) and equilibrated by the same buffer. After washing the column, scFvs were eluted with 250 mM imidazole, pH 8.0, diluted 10 times by ion exchange buffer (10 mM Tris-HCl, 10 mM NaCl, and pH 8.0), and loaded on MonoQ column (GE Healthcare). Elution was performed employing 0 to 0.5 M NaCl gradient. Purified samples of recombinant ScFv were analyzed by Laemmli SDS-PAGE and validated by Western blotting with monoclonal antibodies against c-myc peptide (Novus Biologicals).

#### 2.2.1. Site-Directed Mutagenesis

It was performed using QuikChange Lightning Site-Directed Mutagenesis Kit (Agilent) according to the manufacturer's protocol.

### 2.3. DNA Cleavage Assay

Pure supercoiled form of pBluescript DNA was prepared as described earlier [4]. Various concentrations of purified recombinant scFvs were incubated with 1 *μ*g of plasmid DNA in presence of 1 mM MgCl_2_ for 12 hours at 37°C. Reaction products were resolved in 1% agarose gel and visualized by ethidium bromide staining. Percent of plasmid conversion from supercoiled into circular and linear form was calculated based on the results of densitometry. Acquisition of agarose gel images and subsequent densitometry measurements was done using GelDoc 2000 gel documentation system (Bio-Rad). 

### 2.4. DNA Enzyme-Linked Immunosorbent Assay

Double-stranded DNA fragment of 254 nucleotides was PCR-amplified from genomic DNA of *Fusarium avenaceum* employing 5′-biotinylated primers (5′Bio-CGAACCATCGAGAAGTTC and 5′Bio-CCAGTGGTTAGTGACTGC), synthesized by Syntol. PCR products were treated by phenol-chloroform mixture, pelleted by three volumes of ethanol with subsequent centrifugation (10 minutes at 10000 g) and dissolved in buffer A containing 20 mM Tris-HCl pH 7.5 and 100 mM NaCl. Purification of the DNA fragment was conducted using gel filtration column Superdex G75 (flow rate 0.5 mL/min, sample volume 0.2 mL).

Purified PCR fragment was diluted in buffer A and immobilized on HBC NeutrAvidin Strips (Thermo Scientific) in amount of 1 *μ*g of DNA per well. Unbound DNA was removed by three rounds of washing with buffer A supplemented with 0.02% Tween-20. The surface of the wells was blocked with Superblock Blocking buffer (Thermo Scientific). Recombinant scFvs were applied to the wells in series of concentrations ranging from 0.005 to 5 *μ*g of antibody per well in the same buffer, incubated for 1 hour at room temperature, and washed three times with buffer A. Bound single chain antibodies were hybridized to anti-c-myc monoclonal antibody (Novus Biologicals), which, subsequently to above-described washing procedure, was detected by anti-mouse Fc-fragment peroxidase conjugate and soluble TMB substrate (Thermo Scientific). Reaction was stopped by addition of 10% H_2_SO_4_ and absorbance was measured at 405 nm. Spectrophotometric measurements were done employing Varioskan Flash multimodal plate spectrophotometer (Thermo). 

### 2.5. SPR Measurements

Surface plasmon resonance assay was conducted employing ProteOn XPR 36 analyzer (Bio-Rad). Measurements were carried out in buffer, containing 20 mM Na_2_HPO_4_/NaH_2_PO_4_, 137 mM NaCl, 0.01% Tween 20, and 100 mg/L bovine serum albumin. Biotinylated DNA fragment, obtained as described in the previous section, was immobilized on the surface of NLS ProteOn sensor chip. Interaction of single chain antibodies with DNA was determined at flow rate 75 *μ*L/min. Experimental data was processed using ProteOn Manager Software, and calculation of kinetic parameters was performed based on Langmuir model of protein adsorption.

## 3. Results

In attempt to explore the incidence of DNA-hydrolyzing antibodies among already characterized DNA-specific autoantibodies, we ran BLAST search using sequences encoding VL and VH domains of BV04-01.

BLAST search of homologs of catalytic light chain of antibody BV04-01 revealed relative abundance of identical or highly homologous light chains among sequenced antibodies. At the same time, antibodies contained such L-chains were largely specific to antigens other than nucleic acids. Judging by ELISA data, isolated recombinant L-chain of BV04-01 did not display any DNA-hydrolyzing activity.

Only few antibodies with L-chains highly homologous to that of BV04-01 had specificity to DNA and were listed as autoantibodies. Surprisingly, one of these anti-DNA autoantibodies—MRL-4 [13]—contained VH domain that differed from VH of BV04-01 by only few residues. 

We therefore sought to perform comparative analysis of MRL-4 and BV04-01 with respect to DNA-binding and DNA-cleaving activities in order to obtain new information regarding potential origin of catalytic activity in autoimmune anti-DNA antibodies. 

Genes encoding MRL-4 VH and VL were cloned from corresponding hybridoma cell line and used to construct scFv as it was done for BV04-01 [14]. Recombinant MRL-4 scFv produced in *E. coli* displayed no DNA-hydrolyzing activity using supercoiled plasmid DNA as substrate. Incubation of MRL-4 scFv with various oligonucleotides did not result in detectable DNA-hydrolyzing activity. At the same time, replacement of BV04-01 VL by MRL-4 VL in single chain antibody BV04-01 yielded in antibody with DNA-hydrolyzing activity indistinguishable from that of BV04-01 scFv (87% and 83% of plasmid cleavage, resp. according to densitometric data).

**Figure 1:**
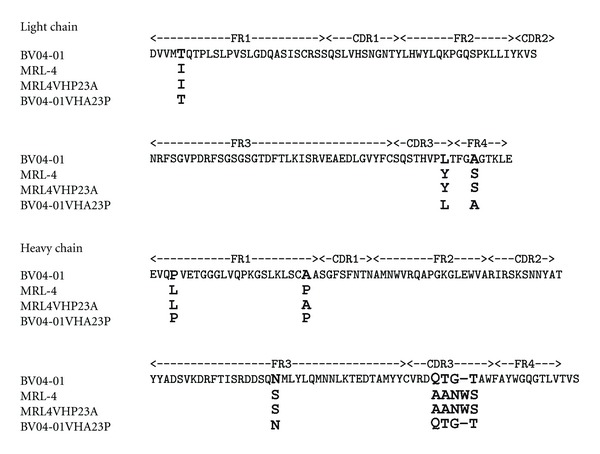
Amino acid sequences alignment of the light and heavy chain variable regions of the antibodies BV04-01, MRL-4, and their mutants.

As it has been inferred from the 3D structure of the complex of BV04-01 Fab fragment with (dT3) [11], and later supported by molecular simulation of the predicted antibody active site and metal-binding pocket [12], formation of the antibody-DNA complex bends DNA, presumably activating one of the phosphodiester bonds. Subsequent hydrolysis and product release necessary for catalytic turnover require conformational changes in the ligand-antibody complex. From previous studies, and in line with hypothesis on antigen-driven nature of anti-DNA autoantibodies [1–3], it is known that some elements of the heavy chain including CDRH3 directly contact DNA in the antibody combining site [11]. At the same time, little is known about structural determinants of conformational dynamics in DNA-hydrolyzing antibodies. Catalysis implies that the enzyme would have been capable of turnover, in other words, for repeated conformational changes accompanying substrate binding, transition state formation, and product release. Therefore, we attempted to identify the antibody elements whose variation can affect conformational rearrangements of the antibody-antigen complex. To do this, we conducted site-directed mutagenesis at selected sites of MRL-4 and BV04-01 antibody chains, which might affect conformation changes in these antibodies, and screened DNA-binding capacity of the resulting mutants by DNA ELISA. Alignment of primary structure for single-chain antibodies BV04-01, MRL-4, and their mutants obtained is presented in Figure 1.

**Figure 2:**
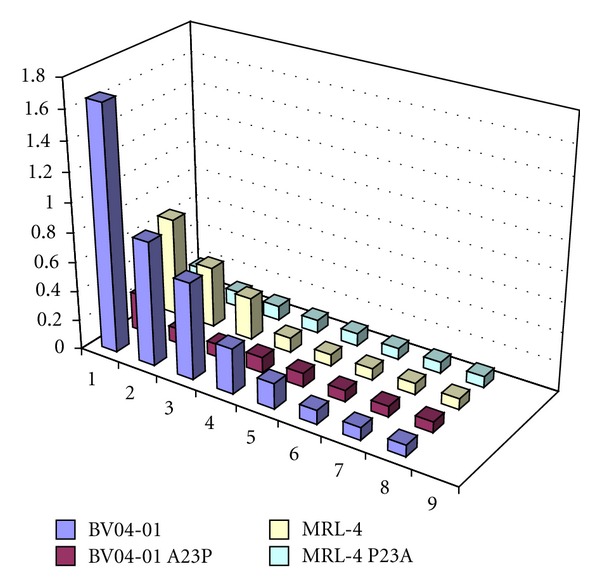
DNA binding by BV04-01 and MRL-4 single-chain antibodies and their mutants assayed by ELISA. Vertical axis represents absorbance measured at 405 nm. Horizontal axis displays amounts of recombinant antibodies per ELISA well: 1: 5 *μ*g, 2: 1 *μ*g, 3: 0.5 *μ*g, 4: 0.1 *μ*g, 5: 0.05 *μ*g, 6: 0.01 *μ*g, 7: 0.005 *μ*g, 8 buffer without antibody. Experimental error for determined absorbance values was calculated within a series of 5-independent ELISA experiments and did not exceed 5% of medium absorbance value.

**Figure 3:**
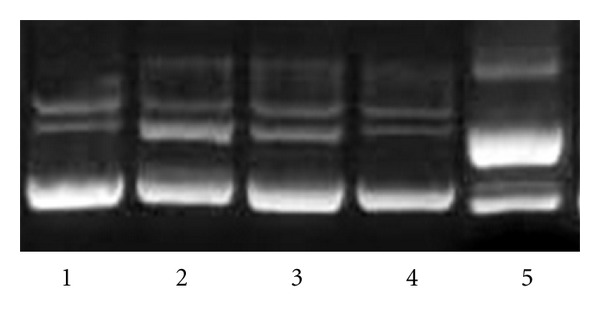
DNA hydrolysis by single-chain antibodies BV04-01, MRL-4 and their mutants. Supercoiled plasmid substrate was incubated overnight with 0.1 mkg of different ScFv in buffer, containing 20 mM Tris-HCl pH 7.5, 100 mM NaCl, and 1 mM MgCl_2_. Lane 1: control plasmid without an antibody; lane 2: plasmid with ScFv mutant BV04-01 VH A23P; lane 3: plasmid with ScFv mutant MRL-4 VH P23A; lane 4: plasmid with ScFv MRL-4; lane 5: plasmid with ScFv BV04-01.

An unusual somatic mutation, L4P (Kabat numbering) is present in the BV04-01 heavy chain. Reversion of this mutation to the germline gene sequence, as well as replacement of proline by alanine, did not affect binding and hydrolysis of DNA by BV04-01 ScFv. Another unusual mutation found in MRL4 is replacement of germline Ala-23 by Pro. Unlike the L4P reversion in BV04-01, reversion of the P23 in the MRL-4 scFv to germline sequence resulted in significant loss of binding capacity of the antibody to DNA. We then asked if acquisition of P23 by BV04-01 VH fragment can cause any changes in its DNA reactivity and analyzed DNA-binding and DNA hydrolyzing activity of A23P mutant of BV04-01 ScFv. Unexpectedly, not only DNA cleaving activity was abolished, but also DNA binding was dramatically decreased due to the A23P mutation in BV04-01 VH, judging by ELISA data. Results of ELISA are presented in Figure 2. Hydrolysis of supercoiled DNA substrate by mutants is shown in Figure 3.

Taking into account significant loss of DNA binding due to mutation effect of Pro to Ala at VH23 of MRL-4, we next analyzed reversion of Pro to the germline Ala in the MRL-4 VH. DNA binding of MRL-4 scFv containing P23A in the VH domain dropped to the level comparable to that of the SCA04-01 VH A23P. To provide accurate quantitative data on changes in DNA-binding capacity of mutant and wild-type variants of MRL-4 scFv and SCA04-01, we analyzed these antibodies using surface plasmon resonance (SPR) technique. SPR data indicated that the biggest decrease in DNA binding efficiency was found in the SCA04-01 VH A23P mutant. Replacement of alanine to proline resulted in approximately 2.5 orders of magnitude drop in *K*_*d*_ and loss of DNA-hydrolyzing activity to the level undetectable by supercoiled DNA cleavage assay. Reversion of P23 in MRL-4 VH to the germline Ala had less pronounced effect, resulting in 20-fold loss of  *K*_*d*_ (Table 1).

## 4. Discussion

Proline is a unique amino acid that can affect the conformation of a protein domain or even an entire protein. Proline switches play significant role in regulation of enzymatic activity in certain synthetic or regulatory pathways [16]. A protein inactive in certain proline conformation is activated by the conformation switch catalyzed by prolyl cis-trans isomerase. There was no data regarding proline switches in antibodies, until Feige et al. discovered proline switch dependent control of CH1 domain folding induced by CH1-CL assembly [17]. Proline-dependent conformation of CDR loops plays important role in shaping of the antigen-binding site.

Kappa-chain cis-proline conformation in the L95 position helps to maintain canonical structure of CDRL3 loop, which is quite stable despite high degree of variability of other amino acid residues in this loop. Other canonical CDR loops in antibodies can also contain Pro residues [18]. Moreover, in the absence of proline, CDR sequence can adopt non-X-proline cis-peptide conformation required for stabilization of the antibody combining site [19]. 

It has been noted that the percentage of proline residues increases by 42% in mice and 50% in human antibodies during affinity maturation [20]. Known proline usage changes are mainly confined to turns and kinked regions, providing overall structure stability increase, while reducing energy needed for the turn stabilization [19]. In anti-DNA autoantibodies, this type of proline mutation (T45P) can be found for example, in the heavy chain of 33.C9 monoclonal antibody [21], where it occurs in the turn region between CDRH1 and CDRH2.

Our data suggests that praline residues occurring via somatic mutation process can play an important role in acquisition and modulation of anti-DNA antibody binding and catalytic activity. In MRL-4, P23 is acquired by somatic mutation resulting in 20-fold increase in the autoantibody affinity to DNA comparing to the antibody variant containing reversion to the germline. Mutation of A23P in BV04-01 heavy chain results in 60-fold reduction of DNA binding and completely abolishes catalytic activity. The 23rd position in the heavy chain is immediately downstream to the invariant Cys residue forming the intrachain disulfide bond. Changes in the flexibility of peptide bond rotation at such position during heavy chain folding may affect final conformation of CDRH1 and potentially cause other long-distance effects on the Fv structure.

Discovery of the naturally occurring mutation that can change binding or catalytic properties of anti-DNA autoantibody by affecting the FV structure rather than specific antibody-antigen contacts raises important questions regarding maturation process leading to development of anti-DNA autoantibodies. Available data shows that DNA-binding activity is gradually acquired by antibodies through somatic hypermutation and antigen-driven selection [1–3, 21]. Replaced amino acids, such as positively charged arginines, are responsible for increasing antibody affinity by forming direct contacts with DNA [22]. Whether antigens represent DNA-protein complexes, DNA mimics, or molecules irrelevant to nucleic acids remains to be determined [2, 10]. At the same time, in a number of cases, no correlation between antigen-driven selection and DNA-binding is observed, for example, when an anti-DNA autoantibody reverted to its germline displays no alterations in DNA binding [23]. 

At present, the physiological significance of P23 mutation in formation of pathogenic anti-DNA autoantibodies cannot be determined due to limited available data. For example, analysis of the literature revealed somatic mutation T24P in the heavy chain of murine cross-reactive anti-pneumococcal/anti-dsDNA autoantibody [24]. Despite extensive analysis of the role of somatic mutations in this antibody in DNA binding affinity and pathogenicity, the role T24P mutation was not studied. Earlier, it has been hypothesized that unusual somatic mutations found in lupus autoantibodies, including those occurring in FRs, do not emerge due to antigen-specific activation, but rather reflect lack of negative selection of B cells acquiring autoreactivity through somatic mutation. This can lead to enhanced survival of B-cells with mutations in antibody genes uncoupled from hot spot targeting [25]. With mutations permissible by the impaired clonal deletion mechanism, the route to the high-affinity anti-DNA autoantibody can be very short, as MRL-4 P23A germline revertant displays very low level of binding to DNA.

Why P23 mutation/reversion has such an opposing effect in otherwise structurally similar antibodies? Depending on the P23 isomerization, the structure of CDRH1 of MRL-4 can differ significantly from that of the BV04-01, thus potentially affecting overall conformation of the antigen-binding site of MRL-4. The latter can be significantly more rigid comparing to that of BV04-01, due to restriction imposed by the presence of P23. Bulky W105 residue in the CDRH3 of MRL-4, lacking in BV04-01, may account for increase in rigidity of antibody-DNA complex, either interacting with DNA via stacking mechanism, or by forming hydrophobic contacts with the light chain. Replacement of P23 by alanine in MRL-4 can introduce flexibility in the entire antigen-antibody complex, increasing motion of certain amino acid residues including, for example, W105, and thus weakening antibody-DNA, or VH-VL interaction, or both.

On the contrary, catalytic activity of BV04-01 necessitates the antibody-DNA complex to maintain substantial degree of flexibility, as catalytic reaction implies initial binding of a substrate to an enzyme, conformational rearrangement required for lowering the reaction energy barrier, and subsequent release of reaction product(s). One can speculate, that introduction of proline at the base of CDR1 can reduce its flexibility, by “freezing” it in certain conformation, thus not only disrupting the contact of His31 with DNA [11], but also impeding overall conformational changes required for successful catalysis of phosphodiester bond cleavage. Interestingly, none of the other DNA-hydrolyzing antibodies with known sequences [26, 27] contain Pro or other potentially conformation-restricting mutations at this or adjacent positions. Comparative analysis between 3D structures of MRL-4, BV04-01, and VH23 mutants thereof can help to understand the mechanism of influence of P23 mutation on DNA-binding and DNA-hydrolyzing activity.

Extensive search for anti-DNA autoantibodies containing this or similar type of mutations and their analysis would help to answer whereas the observed phenomenon is an isolated case or an important route to maturation of DNA-binding and DNA-hydrolyzing autoantibodies contributing to pathogenesis in systemic autoimmune disorders.

## 5. Conclusions

Acquisition of high affinity to DNA in autoantibodies is known to proceed via somatic hypermutation and is believed to be antigen-driven. Corresponding mutated residues are frequently involved in direct contacts with DNA. In this paper, we describe conformation-dependent modulation of DNA-binding capacity of homologous autoantibodies, whereby mutation changing the rigidity of the DNA-binding (MRL-4) antibody Fv or its portion results in significant increase in antibody affinity to DNA. In DNA-hydrolyzing antibody (BV04-01) the same mutation, introduced artificially, decreased binding affinity and abolished catalytic activity. 

Difference observed in the mutation effect hints on the importance of conformation flexibility for the antigen-binding site of a catalytic antibody. Thus, pathways of acquisition of DNA binding can differ in structurally similar antibodies, and this difference may reflect specific conditions under which antibodies become catalytic. Knowledge of such conditions can help to develop catalytic antibodies tailored to target specific epitopes of the antigen of choice. Further structural and statistical data is required to estimate the role of this phenomenon in maturation of anti-DNA autoantibodies.

## Figures and Tables

**Table 1:** Affinity constants of BV04-01 and MRL-4 ScFvs and their mutants determined by SPR.

Wild type and mutant ScFv	ds DNA ELISA*	ds DNA SPR, M^1∗∗^
BV04-01	100 ± 1.21	(1.8 ± 0.31) × 10^−9^
BV04-01 A23P	1.2 ± 0.02	(2.9 ± 0.67) × 10^−7^

MRL-4	100 ± 1.52	(1.7 ± 0.18) × 10^−8^
MRL-4 P23A	4.6 ± 0.61	(8.5 ± 0.84) × 10^−6^

*Binding of wild type and mutant single chain antibodies in solid-phase ELISA with double-stranded DNA. Results for mutants are expressed in relative units by using wild types as standards, which were set to 100 relative units.

**Binding kinetics was analyzed by SPR with double-stranded DNA fragments as ligands and antibodies as analytes and calculated *K*_*d*_ values were presented.

^1^Binding of wild type and mutant single chain antibodies to double-stranded DNA.
